# Supplementary material for: Tissue-Specific Proteome and Subcellular Microscopic Analyses Reveal the Effect of High Salt Concentration on Actin Cytoskeleton and Vacuolization in Aleurone Cells during Early Germination of Barley
Source: Int J Mol Sci. 2021 Sep 6;22(17):9642. doi: 10.3390/ijms22179642 (PMC8431815; doi:10.3390/ijms22179642)
Supplement: Supplementary file 1 [file ijms-22-09642-s001.zip › ZIP/DermSchnur et al_suppl_revi.pdf]

Figures Supplement

Figure Supplement 1:

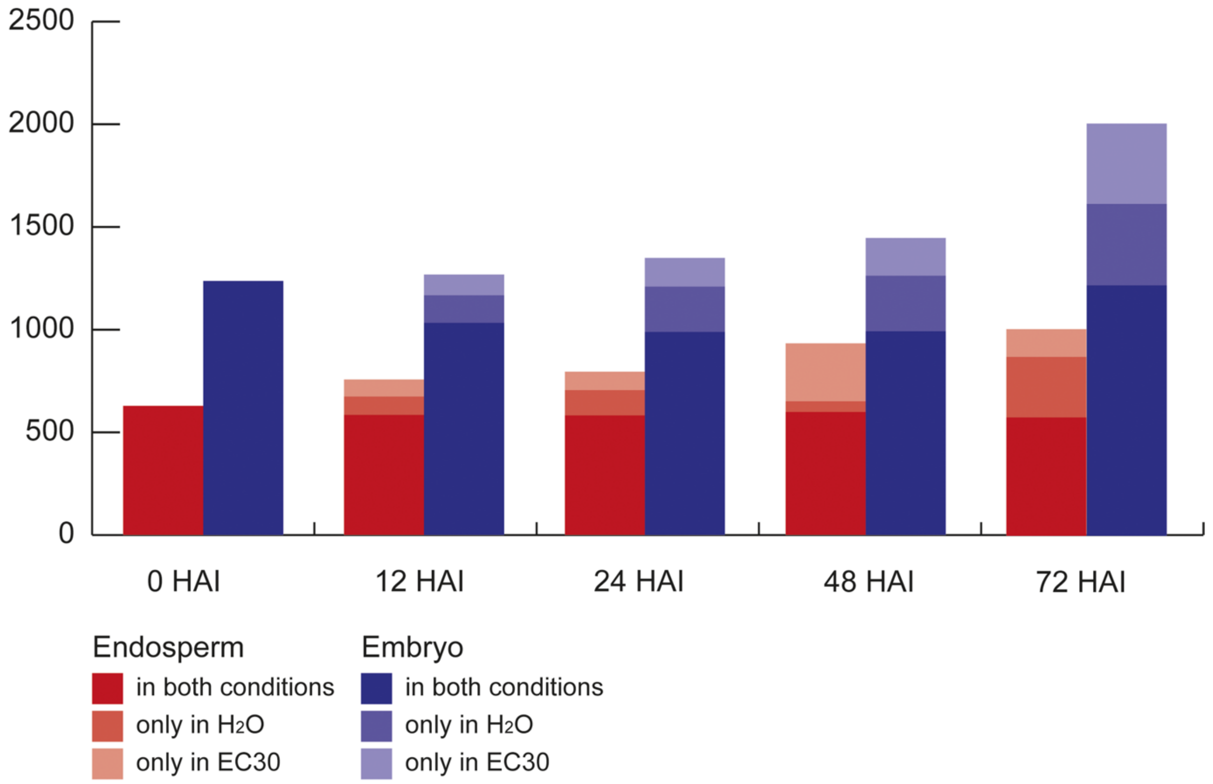

Figure Supplement 1. The number of identified proteins in H_2O and in the EC30 condition.

Figure Supplement 2:

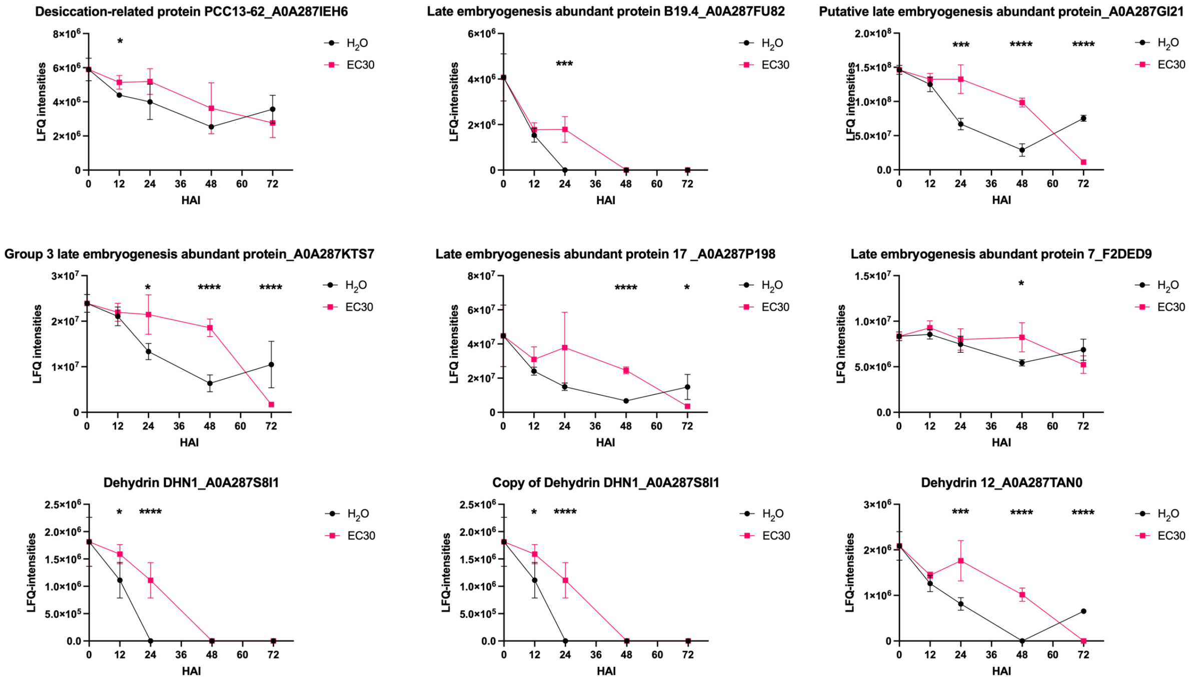

Figure Supplement 2: The protein abundance of LEA proteins at distinct stages during germination within the embryo. Within these points of time, the protein abundance is significantly different between H_2O and EC30. Statistical analyses: multiple-unpaired t-test; significance is indicated with * (p-value ≤ 0.03), *** (p-value ≤ 0.0002), and **** (p-value ≤ 0.0001).

Figure Supplement 3:

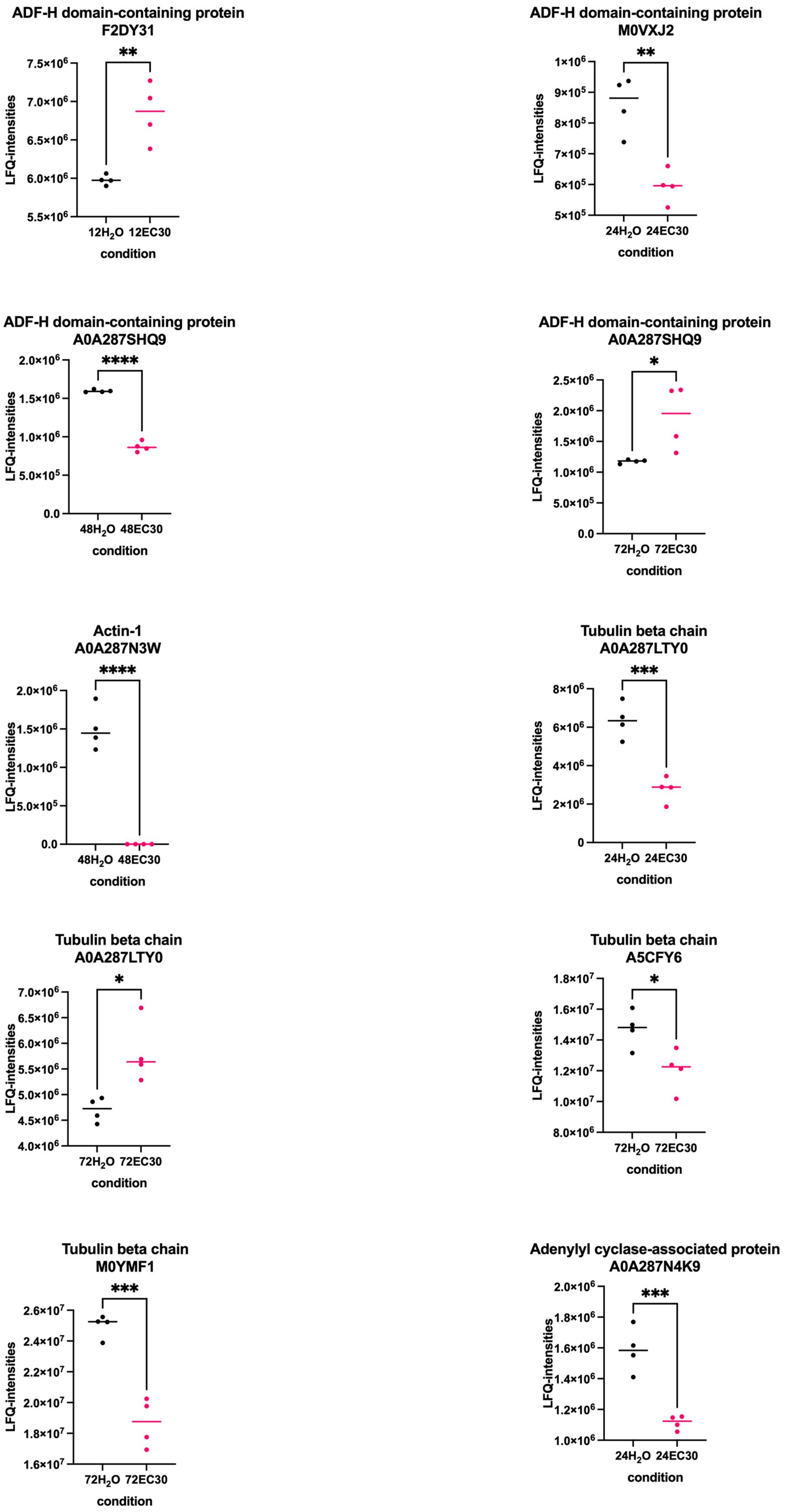

Figure Supplement 3: The protein abundance of cytoskeleton-related proteins at distinct stages during germination of the embryo. Within these points in time, the protein abundance is significantly different between

H_2O and EC30. Statistical analyses: multiple-unpaired t-test; significance is indicated with * (p-value ≤ 0.03), ** (p-value ≤ 0.01), *** (p-value ≤ 0.0002), and **** (p-value ≤ 0.0001)

Figure Supplement 4:

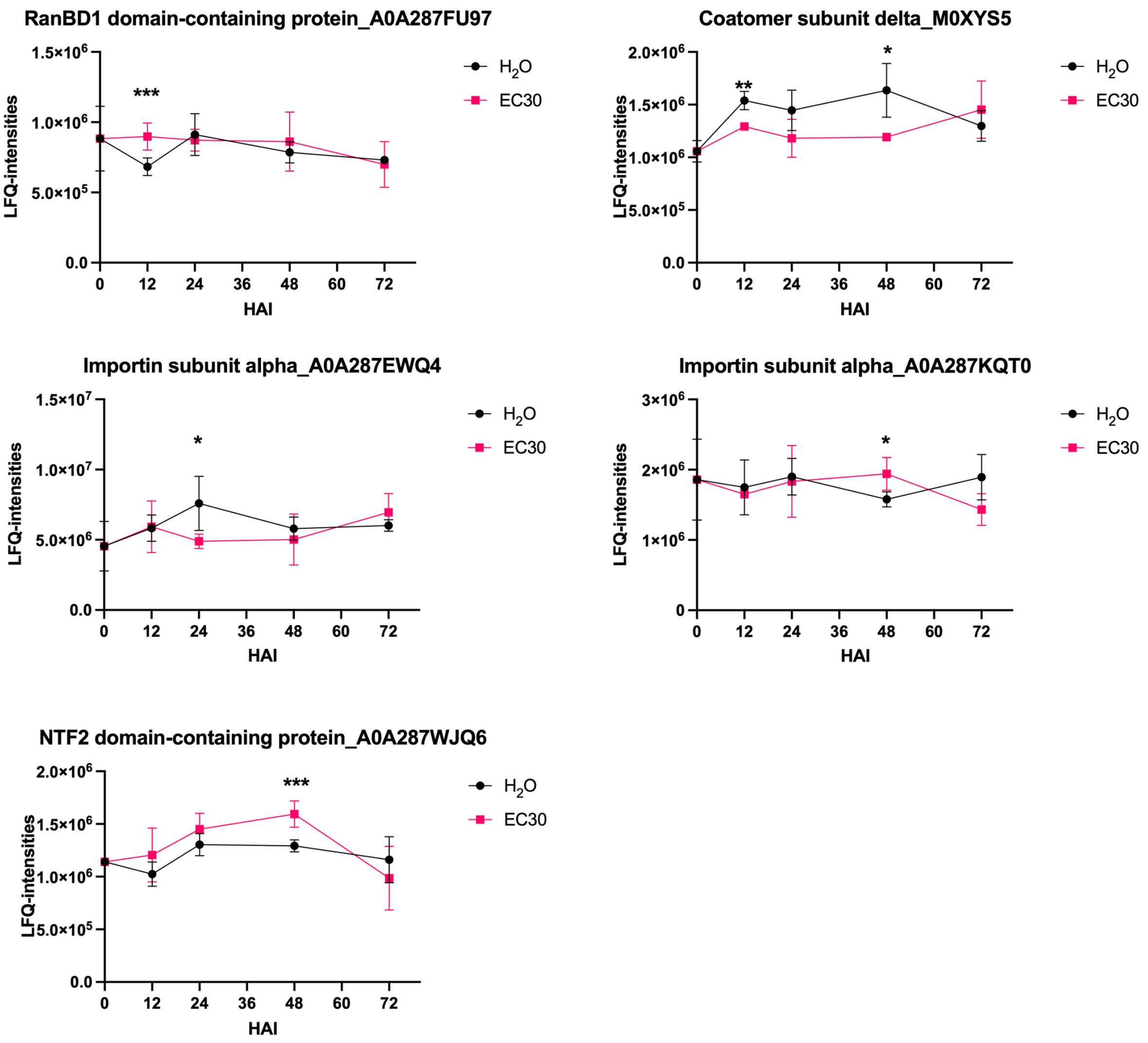

Figure Supplement 4: The protein abundance of protein trafficking-related proteins during germination of in the embryo. Within these points in time, the protein abundance is significantly different between H_2O and EC30. Statistical analyses: multiple-unpaired t-test; significance is indicated with * (p-value ≤ 0.03), and *** (p-value ≤ 0.0002),
